# Girls With Social and/or Attention Deficit Re-Examined in Young Adulthood: Prospective Study of Diagnostic Stability, Daily Life Functioning and Social Situation

**DOI:** 10.1177/10870547231158751

**Published:** 2023-03-13

**Authors:** Svenny Kopp, Karin Susanna Asztély, Sara Landberg, Margda Waern, Stefan Bergman, Christopher Gillberg

**Affiliations:** 1 University of Gothenburg Institute of Neuroscience and Physiology, Göteborg, Sweden; 2 University of Gothenburg Institute of Medicine, Göteborg, VG Region, Sweden

**Keywords:** females, outcome, attention-deficit/hyperactivity disorder, autism spectrum disorder, developmental psychopathology

## Abstract

**Objective:**

Investigate diagnostic stability, daily life functioning and social situation in women diagnosed with ADHD and/or ASD in childhood.

**Methods:**

Prospective 17 to 20-year follow-up study of 100 girls of whom 92 diagnosed in childhood with main DSM-IV ADHD or ASD, and 60 comparison girls. Ninety and 54 of these women were examined (*M* = 27, 4 years old) with semi-structured interviews and questionnaires, close relatives were interviewed.

**Results:**

At follow-up, 89% of women with ADHD or ASD in childhood still met the criteria for either of these diagnoses. Very few women were “in remission.” In 34% the main diagnosis shifted from ADHD to ASD. Women with ADHD and ASD had significantly more disability and unfavorable social situation than comparison women.

**Conclusion:**

Women with ADHD and/or ASD in childhood had impairing problems 17 to 20 years later. Early ADHD changed to ASD in adulthood in some cases. Nearly all with ASD met criteria for ADHD as adults.

Both attention-deficit/hyperactivity disorder (ADHD) and autism spectrum disorder (ASD) are heterogeneous neurodevelopmental disorders (NDDs) with onset early in life. ADHD is defined by impairing symptoms of inattention (ADHD-I), impulsivity/hyperactivity (ADHD-H), or a combination of both (ADHD-C) according to the *Diagnostic and Statistical Manual of Mental Disorders* (4th ed. and 5th ed. [DSM-IV and DSM-5]; American Psychiatric Association [APA], 1994; APA, 2013). ASD is characterized by severe social interaction- communication (SOC) deficits along with repetitive and restricted behaviors and interests (RRBI) according to both manuals. ASD in the DSM-IV compromises the diagnostic categories Autistic Disorder (AD), Childhood Disintegrative Disorder (CDD), Asperger Disorder/Syndrome (AS) and Pervasive Developmental Disorder Not Otherwise Specified (PDD NOS).

The main symptoms of both ADHD and ASD lead to substantial impairments in many important domains of functioning.

Until recently, most ADHD and ASD research has focused on boys and men (Biederman et al., 1999; Dworzynski et al., 2012; Hinshaw et al., 2022; Lai et al., 2015). As a consequence the female presentations have been overlooked both in research and clinical settings (Lai et al., 2022; Lockwood Estrin et al., 2021; Young et al., 2020).

During the past decade, several studies focusing on females have been carried out particularly with regard to ADHD (F. Mowlem et al., 2019; Nussbaum, 2012; Quinn & Madhoo, 2014). A series of very comprehensive longitudinal studies of girls with and without ADHD have been published by the Biederman and Hinshaw research groups (Biederman et al., 2002, 2012; Hinshaw, 2002; Owens et al., 2009). Findings from these two major follow-up studies show that 74% to 77% of females diagnosed at young age with ADHD had persistent and disabling ADHD-symptoms 11 to 16 years later (Owens et al., 2017; Uchida et al., 2018).

Studies on the stability and developmental trajectories of ASD diagnoses in females with ASD are few (Rondeau et al., 2011). However they support the consistency of the condition in both sexes over time (Billstedt et al., 2005; Helles et al., 2015). AD has been shown to be the most stable ASD diagnosis over time and AS and PDD NOS relatively more instable (Kočovská et al., 2013; Rondeau et al., 2011). In comparison of diagnostic outcome between the DSM-IV and DSM-5 in children, PDD NOS was less likely to meet DSM-5 ASD criteria (Harstad et al., 2015). In two review articles on outcome in ASD limited social integration and friendship, lacking in the quality of independent living, poor job prospects and high rates of mental health problems were reported in adulthood (Howlin & Magiati, 2017; Steinhausen et al., 2016). However these outcomes were not stratified for sex, and the role of gender remains uncertain.

While in the past considered to be easily separable disorders, it is now clear that ASD and ADHD are often comorbid with each other, share many genetic factors and show similar functional and structural brain characteristics and cognitive profiles (Rommelse et al., 2010; Visser et al., 2016).

Together with other NDDs (including Language Disorders, Dyslexia, Developmental Coordination Disorder, Intellectual Disability (ID) and Tic Disorders (TD)) ASD and ADHD are sometimes grouped under the acronym of ESSENCE (Early Symptomatic Syndromes Eliciting Neurodevelopmental Clinical Examinations) (C. Gillberg, 2010).

The prevalence of ASD with ADHD for children in clinical studies have been estimated at 53% to 78% (Goldstein & Schwebach, 2004; Sinzig et al., 2009) and in outpatient adults about 40% (Hofvander et al., 2009; Joshi et al., 2013). Autistic traits have been reported from clinical trials in one of three girls with ADHD (Kopp et al., 2010; Uchida et al., 2018). Disability is pronounced in youths with ADHD and autistic traits, compared to those with ADHD only (Joshi et al., 2020). Co-occurrence of ADHD in ASD is associated with ASD more disabling features (Rosen et al., 2018).

## ADHD and Gender

ADHD affects about 5% to 7% of the general population of children (Polanczyk et al., 2014; Thomas et al., 2015) with 1.6:1 male-female ratio (APA, 2013) and 2.8% in adults (Fayyad et al., 2017). The gender difference decreases from 2-3:1 in childhood to 1:1 in adulthood. This decrease can partly be explained by the fact that many women seek neuropsychiatric evaluation as adults independently (without other informants) and that inattentive symptoms, more often diagnosed in girls, but often missed in childhood, persist into adulthood or decline only at a moderate rate (Hartman et al., 2016; Nussbaum, 2012). Hyperactive and impulsive symptoms, more common in boys, decrease more strongly from the age of 12 in both genders (Hartman at al., 2016), but start to increase again in adolescence for girls (Agnew-Blais et al., 2016). It has been shown that hyperactive/impulsive symptoms and behavioral problems more often end up with diagnosis and psychopharmacological treatment compared to other ADHD symptoms in both sexes (F. D. Mowlem et al., 2019). Considering that these symptoms appear less frequently in girls this may be a further explanation for girls with ADHD being missed and misdiagnosed (F. Mowlem et al., 2019) .

Studies focusing on the ADHD symptoms and its effect on women’s psychosocial lives, compared to women without ADHD are few. However adverse outcomes have been reported for young women with ADHD in academic, occupational and social areas (Biederman et al., 2012; Owens et al., 2017; Uchida et al., 2018). Teenage deliveries is more common (Skoglund et al., 2019) as well as intimate partner violence (Guendelman et al., 2016) and sexual victimization (Snyder, 2015). Furthermore the social interaction problems often seen in ADHD influence girls/women more negatively compared to boys/men with ADHD (Kok et al., 2016).

## ASD and Gender

ASD has a population prevalence of about 1% to 1.5% (Elsabbagh et al., 2012; Fombonne, 2005) with a male–female ratio of 1.8 to 3:1 (Kim et al., 2011; Loomes et al., 2017; Mattila et al., 2011). The diagnostic core symptoms (DSM-IV) do not differ considerably between girls/women and boys/men but moderately different cognitive/behavioral phenotypes have been reported in females (Lai et al., 2011; Mandy et al., 2018). Both fewer RRBI symptoms and different social interaction symptoms have been found in females compared to males (Dean et al., 2014; Frazier et al., 2014; Head et al., 2014; Van Wijngaarden-Cremers et al., 2014).

Studies focusing on the ASD symptoms and its effect on women’s psychosocial lives, compared to women without ASD is rare. However two reports have shown greater risk for negative sexual experiences compared to women without ASD (Bargiela et al., 2016; Pecora et al., 2019).There are some suggestions that women with ASD have poorer social outcomes, especially with respect to employment and quality of life than men (Howlin & Magiati, 2017).

## Barriers to Detection in ADHD and ASD and Gender

Similarities between causes of missed or delayed diagnoses in ADHD and in ASD have been found (Lockwood Estrin et al., 2021; F. Mowlem et al., 2019). Several barriers to detection in females with ADHD and ASD have been reported as well as criticism of the diagnostic tools used developed, using predominantly male populations, leading to a lack of sensitivity for female specific ADHD and ASD symptoms (Kopp & Gillberg, 2011; Lockwood Estrin et al., 2021; Ohan & Johnston, 2005). Both ADHD and ASD with normal intellectual ability are diagnosed later in girls than boys and often coexisting behavioral/emotional problems are needed for a diagnosis (Dworzynski et al., 2012; Frazier et al., 2014; Young et al., 2020). Camouflaging behavior has recently been reported among females with both ADHD and ASD leading to an underestimation of their problems (Gould & Ashton-Smith, 2011; Young et al, 2020).

## Aims

The aim was to study diagnostic stability in ADHD and ASD according to DSM-IV established in childhood/adolescence into early adulthood in a clinical group of women. Another aim was to compare the main diagnoses examined according to the DSM-IV and DSM-5. Further, to study overall function level and psychosocial situation compared to a group of women without these diagnoses.

## Methods

The present follow-up study occurred at Time two (T2) after the original diagnostic study at Time one (T1). It was carried out, as part of a larger prospective longitudinal follow-up study of 100 Swedish girls with ESSENCE, the vast majority was diagnosed with ADHD and/or ASD in childhood/adolescence, 17 to 20 years after diagnosis (Kopp et al., 2010). Included was also a comparison group of 60 “non- ESSENCE” girls from the general community.

### Participants

#### Original Study (T1)

##### All Clinic Girls

Between 1999 and 2001, 100 girls aged between 3 and 18 years were assessed for social impairment and/or attention problems at the Child Neuropsychiatry Clinic, Queen Silvia’s Children and Youth Hospital, Gothenburg, Sweden. The investigation was multi-professional and was performed by experienced clinicians. Of the 100 girls participating at T1, 46 received a main diagnosis of ADHD, and 46 a main diagnosis of ASD (AD, AS, CDD, PDD NOS) of whom the vast majority also had “comorbid” diagnosis of ADHD. Of the remaining eight, three were diagnosed with TD and five with other main diagnoses (OMD). Although the inclusion criterion of the study was the absence of ID, 12 girls (10 with ASD) were found to meet the criteria for ID despite having originally been screened ID negative (see reference (Kopp et al., 2010)). All diagnoses were made according to DSM-IV. At T1, using DSM-IV, co-diagnosis of ADHD and ASD was not possible, nevertheless, both diagnoses were examined in all girls and the main diagnosis was used to define the problems most disabling. At T1 the girls diagnosed with ADHD as main diagnosis were significantly older (*M* = 13.0, *SD*3.4) than those with ASD (*M* = 8.8, *SD*4.4) (*p* < .001).

##### Matched Clinic and Matched Community Girls

All clinic girls 7 to 16 years of age with a tested FSIQ ≥ 80 (*n* = 60) were selected and matched for age (±2 months) with 60 randomly selected schoolgirls from the register of a pediatric outpatient clinic in a community in the Göteborg region. Exclusion criteria for the community girls included serious medical illness, ID or having a sibling or parent with a known NDD. Three of 60 community girls were excluded after full assessment at T1. The final community group composed therefore of 57 girls. Among the 60 matched clinic girls ADHD was received in 34 and of them 32% also had autistic trait. Twenty girls received an ASD diagnosis of the 60 matched clinic girls.

#### Follow-Up (T2)

##### Full Clinic Group (FClinG)

During 2015 to 2019, the 100 women who had participated in the original study (T1) when they were ≤18 years of age were invited to participate in a follow-up assessment (T2). Ten of them declined participation. Four further women declined a personal interview but granted permission for a proxy interview with a close relative. Five were not able to participate on their own due to communicative disabilities (all with ASD and ID at T1) but were seen and gave permission themselves or through their legal guardian, for an interview with a close relative (usually the mother). Three women wanted to participate themselves but did not agree to the participation of a relative. We thus received information from both the woman and her relatives in 78 cases, from the woman only in three and from a relative only, in nine cases.

##### Matched Clinic Group (MClinG) and Non-Matched Clinic Group (Non-MClinG)

**Figure 1.**
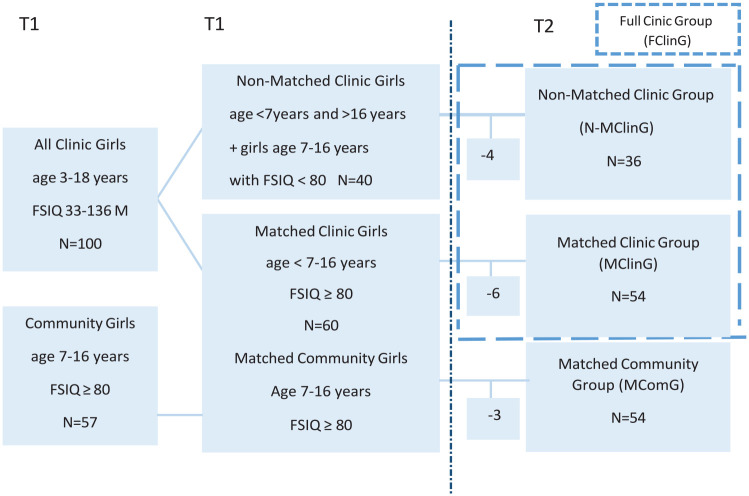
Number of participating girls and women in different study groups at T1 and T2.

All the 90 women in FClinG were included at T2 and were distributed between *MClinG and Non-MClinG*, 54 and 36, respectively (see Figure 1). In the analyses of results obtained in the FClinG at T1, no significant difference was found for Full Scale Intelligence Quotient (FSIQ) between the ASD and the ADHD group (*M* = 95, *SD*22 respectively *M* = 90, *SD*12; *p* = .268). All clinic women with ID were included in the Non- MClinG.

##### Matched Community Group (MComG)

In the MComG 54 of the 57 women participated at follow-up, three were not reachable or declined participation. Two of the women agreed to participate but declined participation of a relative. Altogether, information was obtained from both woman and her relative in 52 cases and from the woman only, in two cases. These 54 women were included in the MComG (Figure 1). After full assessment five women from the MComG were excluded, three women met criteria för ADHD and two for ASD, leaving 49 women for comparison with the MClinG.

### Informed Consent and Ethics

The Central Ethical Review board of the University of Gothenburg has approved the study (file nbr. 855-14, 2015-02-17). All the 144 participating women or their legal guardians, provided written or orally informed consent to participate. Special arrangements were required to ensure that potential participants were fully informed. After the formal invitation was sent, a second simplified version of the letter, was sent as a supplement. Oral information was also provided to ensure that all participants understood what the interview/assessment would entail.

### Measures

All the 144 women were examined with the same instruments according to their ability to participate. The interview included 16 instruments for the women and eight instruments for the first degree relative (see Appendix 1). Most of the clinic women were interviewed during one single session, lasting 4 to 8 hr, but some required two or more sessions. The women in the FClinG were all assessed by one of three physicians (including SK who performed all the evaluations at T1) clinically experienced in the assessment and treatment of NDD. All interviews were conducted in Swedish and all instruments used were translated and previously used in several Swedish studies.

The interview with the relative lasted 2 to 4 hr. When only a relative was interviewed, the assessment was expanded with the semi-structured interviews that were used for the evaluation of the women participating in the study. The woman and her relative were interviewed separately and usually on different occasions. The woman’s current life situation, challenges, and need for support were in particular focus at the interviews of both participants and of close relatives.

Interviews with women and relatives in the MComG were performed by two of the medical doctors and a medical student with a MSc in psychology and lasted 2 to 4 hr. To ensure that the interviews were conducted in a similar manner, six women in the MClinG and six women in the MComG were co-rated and their data discussed in detail in order to improve inter-rater agreement.

#### Instruments Used

##### DSM-IV and DSM-5

Both these manuals were used when checking on diagnostic criteria for ADHD and ASD at T2. For ADHD there are minor differences between these two, there are nine inattentive items and nine hyperactive and impulsive items, in both versions. In DSM-IV for ASD there are four items in the social interaction, four in the communicative (SOC), and four in restricted/repetitive behavior and interest domain (RRBI). In DSM-5 this is modified into three items in the SOC and four in the RRBI domain. To ensure that all criteria were examined, in addition to what emerged during the interview with the woman and her relative, data from the instruments below were used.

##### “Life-Situation-Interview”

Data were collected using a semi-structured interview schedule including 114 questions, in all women who were able to participate by their own. The topics covered in this interview 11 domains. In the actual study we used data from following topics: housing, relations, studies, economy and work/daily activity. We also asked about violence, physically, mentally and/or sexually (rape and/or sexual harassment) and if so, by whom. This type of interview has earlier been used in follow-up studies at our center. However our research team added some “female-oriented” questions specifically developed for this study.

##### WRAADS/WRASS

In the examination process of ADHD, results obtained on the Wender-Reimherr Adult Attention Deficit Disorder Scale (WRAADS) a self-report questionnaire completed by the woman, and the Wender Riktad ADHD Symtom Skala (WRASS)-scale, a Swedish version of the Targeted Attention Deficit Disorder Rating Scale (TADDS) completed by her relative were used. The WRAADS is based on the Utah Criteria (Ward et al., 1993) for ADHD in adults rather than the DSM criteria and was created by assessing the symptoms of adults instead of childhood problems (Marchant et al., 2013). It assesses ADHD symptom severity across seven domains: attention difficulties, hyperactivity/restlessness, temper, affective lability, emotional over-reactivity, disorganization, and impulsivity. It provides several items in each domain rated as present, possibly present, or not present.

##### ASSERT

The Autism Symptoms Self-Report (ASSERT) were used in the diagnostic process of autism, completed by both the woman and her relative (Posserud et al., 2013). The original form has seven items and reported high sensitivity and specificity for suspected ASD for scores ≥8, supporting the validity of the instrument for screening in adolescents. We used a later version with eight items (a question regarding perceptive problems now included), four items targeting social symptoms and four targeting rigid and repetitive behavior. Response options are “not true” (score 0), “somewhat true” (score 1), and “certainly true” (score 2) leading to a score range of 0 to 16 points.

##### ASDI

The Asperger Syndrome Diagnostic Interview (ASDI)—parent version completed by relatives was included in the assessment (C. Gillberg et al., 2001). ASDI is a semi-structured clinical interview for use of adolescents and/or adults with suspected AS/high functioning autism containing 20 items based on the Gillberg and Gillberg criteria for AS (I. C. Gillberg & C. Gillberg, 1989), covering six areas: social interaction, narrow interests, routines/ rituals, speech and language peculiarities, non-verbal communication problems and motor problems.

##### ABAS II

Functional level was assessed by the first degree relative with the Adaptive Behavior Assessment System, second edition (ABAS II) (Harrison & Oakland, 2003). ABAS II includes subscales for communication, community living, functional academics, home living, health and safety, leisure, self-care, self-direction and relationship. Three composite scores are derived from the sum of these 10 scaled scores: cognitive, social, practical, and one overall functioning; general adaptive composite (GAC).

##### GAF

The woman’s Global Assessment of Functioning (GAF) score (Jones et al., 1995) was estimated by the interviewer based on all collected information. GAF scores ranged from 1 to 100 (high score = better function). Separate ratings were made for symptoms and function, and “functional disability” was equated with GAF function scores ≤70. For some ambiguous cases the GAF scores were conjointly discussed with the two authors SK and KA.

##### Perception/Sensory

Problems related to perception/sensory reactions were investigated from the RRBI DSM-5 criteria of ASD and question eight in the ASSERT form; “Do you feel that you/your relative are too sensitive or not at all sensitive to for example, sound, touch, different materials, pain, temperature or smell?.” We reviewed the reports about sensory reactions at T1 in the medical records and in the Autism Behavior Checklist (ABC), perception domain in this follow-up study (Kopp et al., 2010; Krug et al., 1980).

#### Diagnostic procedures

The DSM-IV criteria were applied to establish diagnoses at both T1 and T2, for examining the diagnostic stability. DSM-5 was used for comparison at T2 between the main diagnoses according to variability in criteria across the two manuals. Diagnoses according to DSM-IV and DSM-5 were assigned when both symptoms and impairment criteria were met. Exclusionary criteria were disregarded, as in T1, using the DSM-IV. “Main diagnosis” was used to define the problems considered most handicapping at the time of assessment. If the woman was on medication with stimulants at T2, the diagnosis of ADHD was considered met if clinical interview did not contradict this. ADHD Not Otherwise Specified (ADHD NOS) was defined if five symptom criteria according to DSM-IV or four to DSM-5 were met in one of the three ADHD subtypes together with impairment.

Only full ADHD syndrome is included in the ADHD analyses. PDD NOS was diagnosed if four to five criteria for AD were met, including at least one criterion from the social interaction domain. Autistic traits was “diagnosed” if two to three criteria were fullfilled for AD. Remission for the main diagnoses assigned at T1 was defined if full symptom criteria were not met at T2 and with a calculated functional GAF score >70.

All collected information from the FClinG was evaluated conjointly by the two authors (SK, KA), and from the MComG by KA and SL. Diagnoses of four ambiguous cases were discussed in detail with one of the senior authors (CG). The interviewers were not blind to baseline ascertainment group.

However the majority of young women in the MClinG were assessed by the two interviewer, who did not have met any of the young women earlier and were mostly blind for their main diagnoses.

#### Data analysis

Statistical analyses were calculated with IBM SPSS version 23 software. Continuous variables are presented with means and standard deviations, and *T*-tests were used for comparisons between groups. Chi-square test were used for group comparisons of proportions. All analyses were two- tailed and a *p*-value of less than .05 was considered statistically significant.

## Results

**Figure 2.**
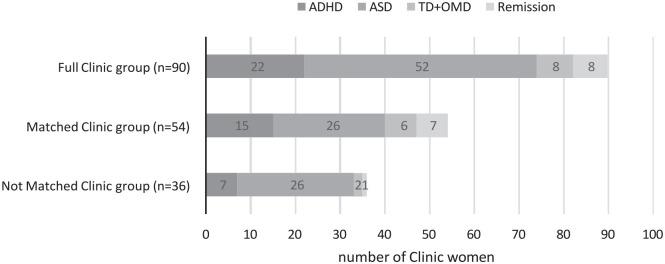
Distribution of main diagnoses and remissions in different study groups at T2 according to DSM-IV.

Results across the two main diagnostic categories, ADHD and ASD (Figure 2), diagnostic stability and perception/sensory problems are reported for the FClinG. Level of functioning and socioeconomic situation is reported for the MClinG, the collapsed ADHD and ASD group (CMClinG) and the MComG.

### Full Clinic Group (FClinG)

At T2 the women in the FClinG were between 19 and 37 years of age, mean age of 27.5 (*SD*4.4). There was a significant difference (*p* = .001) in mean age between women diagnosed with ADHD and ASD as main diagnoses at T1, 29.5 (*SD*3.4) and 25.5 (*SD*4.5). No important difference was found in the distribution of the main diagnoses ADHD and ASD at T1 between the 90 clinic women at T2 and those 10 who were lost to follow up (*p* = .726).

#### ADHD

**Figure 3.**
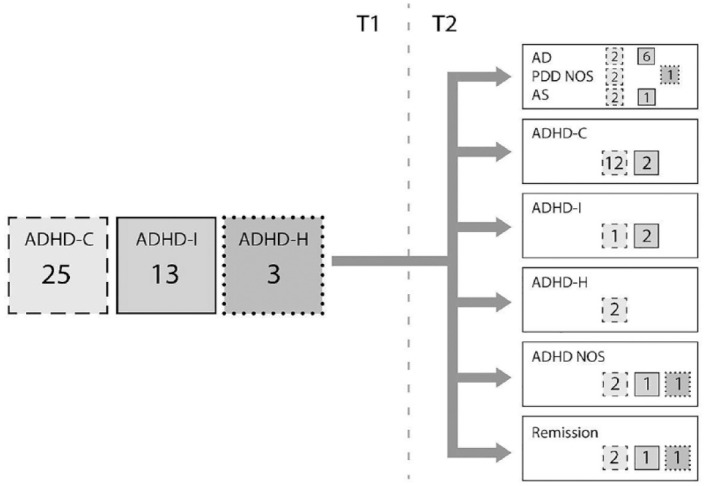
Diagnostic stability and developmental change of main diagnoses from T1 to T2 in the main ADHD group of 41 women.

At follow-up, 74/83 (89%) women diagnosed at T1 with ADHD or ASD still met the diagnostic criteria for either of these main diagnoses and 19/41 (46%) of women with ADHD remained in the ADHD group, and four (10%) fulfilled fewer criteria (ADHD-NOS). Four of the 41 women (10%) were in remission (Figure 3). The combined subtype persisted in 48% from T1 to T2 and was the most stable ADHD condition in the FClinG. The corresponding rate for ADHD-I was 15% and for ADHD-H 0%.

Fourteen women (34%) with a previously main ADHD diagnosis at T1 changed to ASD at T2. Seven of them had also autistic traits at T1 (4 AD, 1 AS, 2 PDD NOS). In the ADHD group without autistic traits at T1 23% met the criteria for ASD at T2 (*p* = .061) and 23% met criteria for ADHD with autistic traits. Twenty two percent from the original ADHD group met an ADHD diagnosis only at T2. All together 54% of the women receiving an ADHD at T2 (10 from ADHD group and two from ASD group) also had autistic traits. The combined subtype was diagnosed in 73% with a main diagnosis of ADHD at follow-up. Only three women met ADHD-I.

At T2 eight more women met a main diagnosis of ADHD according to DSM-5.

#### ASD

**Figure 4.**
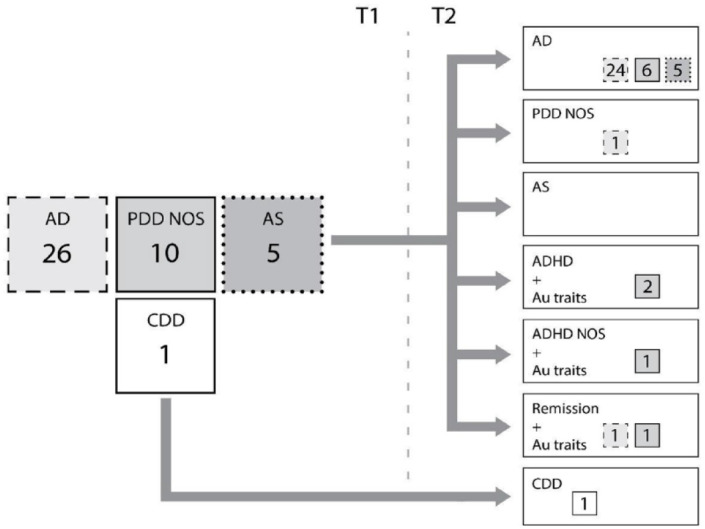
Diagnostic stability and developmental change from T1 to T2 in the main ASD group of 42 women.

At follow-up 88% of women with a main diagnosis of ASD in childhood in the FClinG retained in the ASD group. The vast majority 35/37 (95%) of them fullfilled AD (Figure 4). AD persisted in 24/26 women (92%) from T1 to T2. Six out of 10 women in the PDD NOS group met additional ASD criteria and transitioned to the AD group. Two other women from this group were now diagnosed as ADHD with autistic traits and one with ADHD NOS and autistic traits. All five women with AS transitioned to AD. Two women were in remission, but they did have autistic traits. Full ADHD syndrome was diagnosed in 83% and ADHD NOS in further 13% of the women with ASD at T2, leading to only two women with ASD without any ADHD diagnosis (at T1 79% met full ADHD in the ASD group). The ADHD- subtypes were distributed in the ASD group as follow, ADHD-C (56%), ADHD-I (42%) and ADHD-H (2%)). ADHD-I (42%) was three times as more frequent in the ASD group as in the main ADHD group (14%).

At T2, seven fewer women were diagnosed with ASD in accordance to DSM-5 than to DSM-VI and met instead the full criteria for ADHD with autistic traits. The diagnostic difference was mainly due to more women (67 and 56 women respectively) with the required criteria in RRBI met in DSM-IV than in DSM-5 (one criteria respectively 2).

#### Other main diagnoses (OMD)

Three of seven women retained their diagnoses at T2; one women with TD, one with Mild Intellectual Disability, one with LD NOS. One woman with ADHD NOS changed to ADHD-H at T2. One with Sleep Disorder at T1 was later diagnosed with Restless Legs Syndrome but was now in remission. One woman with Intermittent Explosive Disorder at T1, was diagnosed with ASD and co- occurred ADHD- C at T2.

### MClinG and MComG

ADHD persisted in 14/31 (45%) of women in the MClinG and ASD in 15/18 (83%). The same pattern was seen in the FClinG for these main diagnoses. The diagnostic distribution between the main ADHD and ASD differed strongly from the original assessment at T1 (Table 1). The six women in remission previously diagnosed with ADHD or ASD at T1 emerged from this group. One with TD retained her diagnosis. Four women met an ADHD NOS diagnosis, (three from the ADHD- and one from the ASD- group).

**Table 1.** Main Diagnostic Groups Divided in Main Disorders at T1 and T2.

Assessment time	T1	T2	T2
Diagnostic manual	DSM-IV	DSM-IV	DSM-5
Main disorders in number	*n* = 90	*n* = 90	*n* = 90
ADHD	41	22	30
Combined subtype (st)	25	16	21
Inattentive st.	13	3	5
Hyperactive-impulsive st.	3	3	4
ASD^a^	42	52	45
Autistic disorder	26	44	
PDD NOS	10	4	
Asperger’s disorder	5	3	
CDD	1	1	
Tic disorder	2	1	1
OMD^b^	5	7	6
Remission^c^		8	8

Note. PDD NOS = Pervasive Developmental Disorder Not Otherwise Specified; CDD = Childhood Disintegrative Disorder.

a Autism spectrum disorder with and without ADHD. ^b^OMD: T2 (DSM-VI), Mild Mental Retardation (MMR) (1), Learning Disorder Not Otherwise Specified (LD NOS) (1), ADHD NOS (5). OMD: T2 (DSM-5), MMR (1), LD NOS (1), ADHD NOS (4). ^c^Remission: ADHD (4), ASD (2), TD (1), OMD (1).

At follow-up, two women in the MComG met DSM-IV criteria for ASD, three met criteria for ADHD. These five women were excluded from the MComG in the follow-up analyses, leaving 49 individuals.

### Perception/Sensory Problems

Based on diagnosis in the FClinG of 90 women at T2 sensory reactions were reported significantly less often in the women with a main diagnosis of ADHD compared to ASD (34% vs. 94%; *p* < .001). The same pattern was seen in the MClinG between the two main diagnostic groups (ADHD 25% vs. ASD 96%). All women with ADHD and perceptual problems at T2 also had autistic traits. In the entire ASD group at T1 93% reported sensory reactions leading to a high consistency for sensory problems from T1 to T2 (81%). Fifteen women with main ADHD, reported no sensory problems. In the 14 cases with a change from ADHD at T1 to ASD at T2, 78% already had reported sensory problems at the first assessment time. Very few women in the matched community group reported perceptual problems compared to two of three in the collapsed ADHD + ASD group (*p* = .001).

### Level of Functioning (Table 2)

**Table 2.** Functional Characteristics in the MClinG of ADHD, MClinG of ASD, the Collapsed Diagnostic Group, CMClinG, and MComG at T2.

	ABAS II^b^	MClinG ADHD		MClinG ASD			MClinG ADHD^a^+ASD^a^		MComG		
		*n* = 13		*n* = 25			*n* = 38		*n* = 47		
	GAF f^c^	*n* = 15		*n* = 26			*n* = 41		*n* = 49		
		Mean	SD	Mean	SD	*p*	Mean	SD	Mean	SD	*p*
ABAS	Cognitive	80.9	24.7	72.9	21.5	.31	75.6	22.6	108.2	4.7	<.001***
Index	Social	84.5	21.1	72.1	19.6	.08	76.4	20.8	107.1	7.7	<.001***
	Practical	96.8	21.4	90.6	18.3	.36	92.7	19.8	117.6	4.5	<.001***
	GAC^d^	88.8	23.5	79.2	18.3	.17	82.5	20.4	116.4	5.7	<.001***
GAF f^c^		63.2	14.3	48.9	10.7	.001	54.2	13.8	82.8	9.8	<.001***

Note.^a^DSM-IV main diagnoses, ^b^ABAS: Missing data = in the ADHD group (*n* = 1); ASD group (*n* = 2); CMClinG ADHD+ASD group (*n* = 3); MComG (*n* = 2). GAF^c^: No missing data; function scores ^d^General Adaptive Composite (GAC).

* *p* = statistically significant; *p < .05;**p < .01; ***p < .001).

The level of functioning in daily life in all women with ADHD *(n* = 41) and with ASD (*n* = 42) diagnosed at T1 was measured to study the outcome at T2. The ADHD and the ASD group did not differ significantly in FSIQ (*p* = .270) measured at T1 (ADHD, *M* = 95, *SD* 12, ASD, *M* = 90, *SD* 22). However the functional ability was lower in the ASD group (nine women with ID) compared to the ADHD group on ABAS-II, with a significant difference for practical index (*p* = .002). Nearly all women with ASD also had “comorbid” ADHD.

As described in Table 2 comparisons were made between the two main diagnostic groups and the MComG (all with FSIQ ≥ 80). The ASD group did not differ significantly from the ADHD group on any of the ABAS II’s domains although an overall lower functional ability was reported and a statistical trend was found in the domain of social functioning (*p* = .08) with lower scores for the ASD group. Strongly significant differences were found on the results from ABAS-II between the collapsed ASD and ADHD group and the MComG group with neither diagnosis.

The global functioning (GAF) score differed between the two diagnostic groups with significantly lower GAF scores in the MClinG with ASD (*M* = 49, *SD*11) compared to the ADHD group (*M* = 63, *SD*14) (*p* = .001). The collapsed group of ADHD + ASD differed significantly in comparison to the MComG in the GAF scores. The six women in remission scored highest on the GAF (*M* = 78).

### Socioeconomic Situation

#### Social situation

As described in Table 3., there was a significant difference between the matched ADHD + ASD group and the MComG in the reported need of professional support for living (*p* = .006). All but one of these clinic women were diagnosed with ASD. Living with a partner (all male) were reported in 77% of women in the MComG and, in 41% of the MClinG with NDD (*p* = .002). The majority of women who never had a relationship lasting more than 3 months is found in the ASD group.

**Table 3.** Socioeconomic Situation in the MClinG of ASD, MClinG of ADHD and MComG and Comparison Between the Collapsed Diagnostic Groups (CMClinG) and MComG at T2.

	MClinG ASD	MClinG ADHD	ASD vs. ADHD	MComG	CMClinG ASD+ADHD^a^ vs. MComG
	*n* = 26 (%)	*n* = 15(%)	*p*	*n* = 49(%)	*p*
Social situation					
Living with parents	3 (12%)	0		2 (4%)	
Supported housing	2 (8%)	0		0	
Professional home support	5 (19%)	1 (7%)	.224	0	.006**
Living with partner	10 (38%)	7 (47%)	.812	36 (77%)	.002**
Having children	5 (19%)	7 (47%)	.083	15 (31%)	.890
Abortions *n* = 1–2	5 (19%)	8 (53%)	.038*	9 (16%)	.142
Perceived social isolation	13 (50%)	6 (40%)	.536	7 (14%)	.002**
Highest completed studies					
Elementary school	7 (27%)	5 (33%)		0	
Schooling beyond elementary	16 (62%)	8 (53%)		24 (49%)	
University degree	1 (4%)	1 (7%)	.686^c^	23 (47%)	<.001^c^***
Other	2 (8%)	0		2 (4%)	
Source of income					
Salary	8 (31%)	7 (47%)	.309^d^	34 (70%)	.002^d^**
Social security	1 (4%)	1 (7%)		0	
Sickness benefits	3 (12%)	1 (7%)		0	
Disability allowance	10 (38%)	2 (13%)		2 (4%)	
Other^b^	4 (15%)	4 (27%)		13 (26%)	
History of abuse					
Sexual abuse	15 (58%)	7(47%)	.495	22 (46%)	.408
Raped	12 (46%)	5 (33%)	.422	7 (14%)	.004**
Physically abused	13 (50%)	10 (67%)	.300	8 (17%)	<.001***
Mentally abused	21 (81%)	14 (93%)	.273	16 (33%)	<.001***

Note. ^a^ADHD +ASD diagnostic collapsed group; ^b^Study grants, savings or living on partner income; ^c^University degree vs all other education; ^d^Salary vs all other incomes.

*p* = statistically significant (*p<.05; ** *p*<.01; ***p<.001).

There was a statistical trend (*p* = .083) found in comparison between the ADHD group and the ASD group in children that they had, most children lived with mothers in the ADHD group. One of two women with ADHD had performed 1 to 2 abortions and only women with ADHD had went through more than one abortion (*p* = .038).

In the MClinG 50% respectively 40% stated that they felt socially isolated, a significant difference (*p* = .002) compared to 14% in the MComG.

#### Academic performance

Significant differences were found in the self- reports of highest completed academic achievement between the ADHD and ASD groups and the community group. Among women with ADHD and with ASD one-third reported no education beyond the first 9 years. In contrast, all women in the MComG continued their education, 47% of them had a university degree and 5% in the MClinG. Nearly all clinic women (95%) with ADHD and ASD reported that they had received some type of support during their schooling.

#### Job performance

There were major significant differences in how to provide for oneself between the MClinG and the MComG (*p* = .002) (Table 3). Fiftyfour percent of women with ASD and 27% with ADHD were supported from the society compared to 2% in the MComG. None in the MComG were provided for by their partners, but two with ADHD and two with ASD in the MClinG.

#### Exposure to violence

A high rate of being exposed to sexual abuse were self-reported in all study groups. However rape was significantly more frequently reported in the collapsed MClinG with main ADHD and ASD compared to MComG (*p* = .004) as well as physical and mental abuse (*p* < .001). The mental and physical abuse in the ADHD and ASD groups were mainly carried out by family members or peers and women with ADHD reported the highest degree of exposure.

## Discussion

In this 17 to 20-year follow up of 90 women diagnosed with ADHD and/or ASD in childhood or adolescence the majority still met full criteria for either ADHD and/or ASD. Very few women were in remission. Nearly all the clinic women with ASD also had co-occurrence of ADHD. A change from ADHD in childhood to ASD in adulthood was not uncommon. Perceptual problems were reported in the vast majority of women with ASD.

Girls with ADHD and/or ASD diagnosed in childhood had a significant degree of impairing difficulties in early adult adulthood. It would seem, on the basis of our findings, that ASD with co-occurrence with ADHD might lead to even more pronounced impairment than ADHD in itself. Women with these childhood diagnoses to a large extent had low educational achievement and a higher need for financial and practical support for their livelihood. They appeared to be at greater risk of being subjected to mental and/or physical violence, unwanted pregnancies and sexual abuse.

### Diagnostic Stability and Diagnostic Development

The persistence of any of the two main diagnoses ADHD and ASD was seen in 89% of the women diagnosed in childhood/adolescence. ASD was the most stable diagnosis of the two main diagnoses ADHD and ASD according to DSM-IV and the diagnostic stability was seen in 88% of all women with ASD. For AD the stability was even higher (92%). Our findings correspond to a meta-analysis, which indicated that AD was stable in 76% over time and PDD NOS was less stable (35%), however all these studies included young children (Rondeau et al., 2011). Other outcome studies have confirmed high diagnostic stability for the AD diagnosis (Howlin & Magiati, 2017; Steinhausen et al., 2016). No women with AS diagnosed at childhood (T1) persisted, all (five) ended up in AD. However the group was small. In the Faroe Islands follow-up study of ASD, AS was the least stable diagnosis (Kočovská et al., 2013). In our study no-one of the 10 cases with PDD NOS diagnosed at childhood persisted into adulthood and the majority ended up in the AD group, while a minority either met an ADHD diagnosis or remitted. The same pattern of diagnostic development was reported by Rondeau et al. (2011).

Our finding of 46% of diagnostic stability from T1 to T2 for a full ADHD diagnosis and 10% for ADHD NOS (subsyndromal ADHD) is in line with the two major female ADHD longitudinal studies (Owens et al., 2017; Uchida et al., 2018). In the Massachusetts General Hospital Longitudinal Studies 77% of boys and girls with ADHD continued to display full (35%) or subsyndromal ADHD (42%) as adults (Uchida et al., 2018). The Berkley Girls with ADHD Longitudinal Study reported 57% of diagnostic persistence from childhood to young adulthood and 74% for symptom-based diagnostic persistence beyond childhood (Owens et al., 2017). Few women in our follow-up study were in remission, in the ADHD group 10% and in the ASD group 5%, over a period of 17 to 20 years. Full syndrome remission for females with ADHD has been reported as higher in both the Biederman and Hinshaw groups, 65% respectively 67%. In our assessment at T2 there was a diagnostic change from ADHD to ASD in 34% of the women diagnosed early with ADHD. Neither of the two other longitudinal studies reported similar diagnostic transition, they reported however a greater proportion of women, compared to us, with functional and symptomatic impairment caused by the ADHD symptom. These different findings between our studies might be caused by the recruitment of the sample of girls included in the original studies. Our participating girls at the first assessment were referred to a specialist clinic.

To our knowledge, we have not found any longitudinal study in females with ASD. The male follow- up study in AS (Helles et al., 2015) reported that 22% of the men were in remission and did not meet criteria for any ASD diagnosis.

The combined subtype had the highest persistence (48%) of the ADHD conditions and was also the most common subtype both in the main ADHD group and in ASD with co-occurrence ADHD. The persistence for ADHD-I was only 15%. The Berkley Girls Study mentioned above concluded that a comparatively larger proportion of girls with ADHD-C retain their diagnosis compared to ADHD-I (Hinshaw et al., 2012). These findings that many women with a primary diagnosis of ADHD in childhood were diagnosed with ADHD-C in adulthood, contradicts the previous perception that ADHD- I is the dominant type among females (Agnew-Blais et al., 2016; Nussbaum, 2012). Teicher et al. (2012) also found that adults with ADHD still present with marked overactivity and proposed that the fading of hyperactivity in adolescence and young adulthood is somewhat overestimated. However the inattentive subtype was diagnosed three times more often in the ASD + ADHD group than in the main ADHD-group. Based on our findings, the question arises as to whether some of the girls diagnosed with ADHD-I would actually meet the criteria for ASD.

The high change from ADHD to ASD in one of three women from the original study to assessment two was not expected, even though half of them had autistic traits already at T1. For some of these women there had been suspicion about ASD at the first assessment time. Lockwood Estrin et al. (2021), have compiled the barriers for both girls and women to be diagnosed with ASD, and identified several areas in the family, in society and within the clinic. Especially two behavior characteristics have shown a tendency to confuse the clinician when diagnosing ASD: firstly the instrument used, which not has been developed for girls and the items for social interaction and friendship are possibly not sufficiently adapted, secondly the girls with ASD and extreme hyperactivity with or without aggressive outbursts, tend to conceal the core symptoms of autism.

We found coexisting ASD and ADHD, in 83% and several women with ADHD also had autistic traits (54%). These symptoms partly overshadow each other and cause diagnostic difficulties (Kopp et al., 2010). These results underscore the need for assessment of both types of problems/ diagnosis in order to treat adequately.

There was a trend toward more severe ASD diagnosis over time with an increase in the number of girls fulfilling all 4 DSM-IV SOC criteria at T2 compared to T1. Our findings for adult women expand on those from the ALSPAC birth cohort study that found an escalation of ASD across adolescence that was greater in females compared to males (Mandy et al., 2018; Riglin et al., 2021).

### Perception/Sensory Problems

Nearly all women with ASD in the FClinG had perception/sensory problems at T2 and all with reported perceptual problems at the first assessment retained their sensory problems at T2. Sensory reactions were not reported in the main ADHD group without autistic traits, but most of the women with ADHD and autistic traits reported such problems. The findings are consistent with previous work showing that the majority of all children with ASD have had sensory problems remaining into adulthood (Crane et al., 2009; Leekam et al., 2007). Constantly feeling one’s body and surroundings in sensory terms could be a partial explanation for the fatigue problems that individuals with ASD report. In our previous study, it was found that fatigue is a very common problem in this group (Asztély et al., 2019).

Neufeld et al. (2021) confirms that different sensory processing is not specific to ASD, but ASD is a strong predictor of certain sensory processing alterations. In one study females with ASD reported more lifetime sensory symptoms than men (Lai et al., 2011). Perceptive peculiarities have been stated by autistic individuals as one of their core symptoms (Chamak et al., 2008). Our findings and previous findings suggest that altered sensory experiences should receive extra attention when investigating NDD and possible ASD.

### Level of Functioning

In the MClinG of women the significantly larger general impairment of functional level compared to the women in the MComG, remained from the first assessment at T1 (Kopp et al., 2010). This pattern has been reported from both ADHD and ASD (all male) populations (Helles et al., 2015; Owens et al., 2009). There was a clear difference in the MClinG between women with ADHD and ASD, where the ASD group (nearly all with co-occurred ADHD) ends up lower in GAF scores. However, proxy-rated ABAS score did not significantly differ in women with ADHD and those with ASD (nearly all with ADHD), although there was a statistical trend for greater social disabilities in the ASD group. A weakness with ABAS-II is that the informants report whether their relatives are able to perform certain tasks, not whether they actually perform them without support. Other researcher have noted this problem (Kraper et al., 2017). The differences between the diagnostic groups, with even lower GAF scores for the ASD group remained since childhood, but overall the GAF scores were lower in T1 (Kopp et al., 2010). Possible explanations for the more highly rated GAF scores might be adaptive development factors according to age or to the development of better camouflaging behavior (Quinn & Madhoo, 2014).

In this study the FClinG women with a main ADHD diagnosis or co- existent ADHD in T1 were offered treatment with stimulants and psychoeducation, after the study, during their upbringing. In our previous report we found that one-third of these women today had ongoing treatment with stimulants (Asztély et al., 2019). In previous follow-ups of persons with ADHD, differences in functional level remained even though treatment with drugs and psychoeducational measures according to the golden standard of the time were offered (Molina et al., 2009).

### Psychoeconomic Situation

The MClinG women with ADHD and/or ASD differed significantly from the women in the MComG in multiple domains. The important differences found in our study in social situation, educational level, source of income and personal security in life have earlier been described for mostly clinically referred women with ADHD (Owens et al., 2017; Uchida et al., 2018). A population- based study reported recently that childhood ADHD is associated with adverse adult outcome in many areas which accords with the findings of the present study (Harstad et al., 2022). We have not been able to find any previous study on psychosocial status with regard to highly functioning women with ASD. Most of the women with ADHD and ASD in the MClinG had moved from their parents to their own household. Women with ASD in particular but also some with ADHD were in need of professional support in their home. The women in the MComG lived to a significantly greater extent in a relationship. There were differences between the groups of women regarding the number of pregnancies and children that they had, it was more common for women with ADHD compared to ASD and to the women in the community group. While sexual abuse was experienced by half of the women in the MClinG and a similar proportion in the MComG, being subject to rape was more common in the group of women with ADHD and/or ASD. There are several studies of adolescence with ADHD regarding risky sexual behavior, increased risk of teenage pregnancies and for adolescents with NDD, an elevated risk for being sexually abused (Hua et al., 2021; Ohlsson Gotby et al., 2018; Skoglund et al., 2019).

Studies regarding exposure to sexual abuse in adulthood appear to be sparse. There are some recent work who has pointed out the increased risk of sexual victimization that women with ADHD and ASD are exposed to (Guendelman et al., 2016; Pecora et al., 2019; Snyder, 2015). The background to this is most likely multifactorial with difficulties in interpreting social situations as well as lower self- esteem and more academic failures seen as contributing factors (Bargiela et al., 2016; Guendelman et al., 2016).

Nearly half of the women with ADHD or ASD reported an experience of involuntary social isolation, whereas this was infrequently reported in the MComG. Social difficulties are a core symptom of autism, and social problems in a person with ADHD may reflect social maladjustment due to impulsivity and inattention toward social codes (Kok et al., 2016). However there is a difference between choosing to be alone and involuntary ending up in loneliness. In recent years, social isolation regardless diagnosis, has received increasing attention as a risk factor for poorer physical and mental health in all age groups (Hämmig, 2019).

While the vast majority of women in the MComG supported themselves with a salary/study grant, a fourth of those with ADHD and more than half of the women with ASD had some type of social welfare grant. A partial explanation in addition to their disabling symptoms, might be the large difference in completed education. It is currently challenging to be hired in Sweden without schooling beyond elementary school. Secondary school appears to be necessary. Becoming dependent on income through social insurance, early in life, means a life in relative poverty, this too being a factor contributing to poorer physical and mental health (Brisson et al., 2020).

Large and significant differences were seen when regarding exposure to physical or mental abuse from peers, partners or strangers. Other work by Guendelman (2016) presented the conclusion that young women with persistent ADHD had a higher risk of experiencing intimate partner victimization than young women without this diagnosis. Differences in violence experienced, more pronounced in the ADHD group, were already seen at T1 (Kopp et al., 2010), and even reported in the first female ADHD study by Hinshaw (2002).

### Strengts and Weaknesses

Although the number of participants in our study is relatively small, one of the strengths is that it had a clinical approach from the beginning. At T1 the group was well characterized and examined with rigorous diagnostic criteria. At T2 the information was obtained from the women, their first degree relative, and questionnaires as well as through observations by the researchers during the interviews. This procedure has previously been deemed necessary to obtain a complete picture of the current situation (Sibley et al., 2016). The participation rate is high for a longitudinal follow-up study. We had sufficient information to allow for diagnosis of 90% of the women in the original group of clinic girls.

Available data was the decisive factor for the number of participants and for example, the wide age span, which is a limitation to the study with regard to the changes in diagnostic status observed and presented.

Clinical samples generally include more severe cases than population samples, and due to known referral bias especially among girls, they may not fully provide the whole picture regarding the transition into adulthood among girls with ASD and/or ADHD (Lockwood Estrin et al., 2021; F. Mowlem et al., 2019). The community group was originally recruited from a pediatric outpatient clinic, which possibly distinguishes them from a purely community recruited group.

The fact that one of the researchers, who was responsible for the assessments in T1, also participated in the evaluations in T2 can be seen as a weakness. The interviews were not blinded. To avoid bias as much as possible, the majority of the women and their relatives in the matched groups, were examined by colleagues who did not participate in T1. When conducting the study it was a strength, that good knowledge of the families facilitated the contact. It also made it easier to adapt the assessments where the women could not participate in the interviews (according to severe autism with ID and/or language disabilities) and the parent became the source of information. This fact most likely also contributed to the high retention in participation.

Diagnoses are to be considered as the best estimates of the condition. As in clinical work, it is difficult to get distinct responses from persons with neuropsychiatric symptomatology, and disabilities are often underreported (Sandra Kooij et al., 2008). In the research- setting, interviews more commonly take place under orderly circumstances in premises suitable for the purpose, well timed, one to one, which may cause a certain bias.

## Conclusion

Most of the women with ADHD and/or ASD diagnosed in childhood/adolescence, still met the criteria in early adult life for these. A “transition” from ADHD in childhood to ASD in adulthood occurred in some cases, both from the ADHD-C and ADHD-I childhood groups. Co-occurrence of ADHD and ASD was extremely common. Perceptual/sensory problems occurred in almost everyone with ASD and frequently in women with ADHD and autistic traits. Daily adaptive functioning were impaired in both matched clinic diagnostic groups, however even more so practically in the full ASD group. The low GAF scores in both groups indicate the burden of impairments and the need for support. Further, findings indicate an increased risk of violence and sexual abuse/exploitation compared to peers in the community.

It seems important that when ADHD or ASD is suspected in a girl or an adult woman, the presence of coexisting ADHD and ASD should always be investigated. Clinicians should also plan for continued support that takes into account potential changes in the symptom profile and social situation that may occur in early adulthood.

Further research should be done to better understand the consequences of living with ADHD and/or ASD in adulthood. With better knowledge, medical care, community, educational institutions and employers would be better at detecting the unmet needs of these women and provide more functional support.

## Supplemental Material

sj-docx-1-jad-10.1177_10870547231158751 – Supplemental material for Girls With Social and/or Attention Deficit Re-Examined in Young Adulthood: Prospective Study of Diagnostic Stability, Daily Life Functioning and Social Situation Click here for additional data file. Supplemental material, sj-docx-1-jad-10.1177_10870547231158751 for Girls With Social and/or Attention Deficit Re-Examined in Young Adulthood: Prospective Study of Diagnostic Stability, Daily Life Functioning and Social Situation by Svenny Kopp, Karin Susanna Asztély, Sara Landberg, Margda Waern, Stefan Bergman and Christopher Gillberg in Journal of Attention Disorders
