# Supplementary material for: Girls With Social and/or Attention Deficit Re-Examined in Young Adulthood: Prospective Study of Diagnostic Stability, Daily Life Functioning and Social Situation
Source: J Atten Disord. 2023 Mar 13;27(8):830–46. doi: 10.1177/10870547231158751 (PMC10201085; doi:10.1177/10870547231158751)
Supplement: sj-docx-1-jad-10.1177_10870547231158751 – Supplemental material for Girls With Social and/or Attention Deficit Re-Examined in Young Adulthood: Prospective Study of Diagnostic Stability, Daily Life Functioning and Social Situation [file sj-docx-1-jad-10.1177_10870547231158751.docx]

Appendix 1

**Instruments used in interviews with participants:**

1. Semi-structured interview concerning Life situation

2. Semi-structured interview concerning Medical history

3. SF-36; A 36-Items Short Form Health Survey

4. Neuropsychiatric status, a checklist of symptoms completed during the interview.

5. To document experienced pain, a template with 18 predefined body region was used.

6. WRAADDS; Wender-Reimerr Adult Attention Deficit Disorder Scale

7. MINI; Mini International Neuropsychiatric Interview

8. BDI-II; Beck Depression Inventory

9. MANSA; Manchester Short Assessment of Quality of Life

10. BADS; Behavioural Assesment of Dysexecutive Syndrome

11. LHA-R; Lifetime History of Aggression Scale- Revised

12. ASSERT; Autism Symptoms Self Report

13. DSM- IV; ADHD criterion (same as in DSM-5)

14. DSM-IV; Autism criterion

15. DSM-5; ASD criterion

16. GAF; Global Assessment of Functioning score

**Instruments used in interviews with relatives:**

1. Semi-structured interview regarding need for assistance and given support

2. Semi-structured interview concerning their own social and health situation

3. WRASS 2; Wender-Reimerr adult ADHD scale

4. DSM-IV; ADHD criterion (same as in DSM-5)

5. DSM-IV; Autism criterion

6. DSM-5; ASD criterion

7. ABAS-II; Adaptive Behaviour Assessment System, second edition

8. ASDI; Asperger Syndrome Diagnostic Interview
